# Sarcoidosis following SARS‐CoV‐2 infection: Cause or consequence?

**DOI:** 10.1002/rcr2.955

**Published:** 2022-04-27

**Authors:** Esther Palones, Virginia Pajares, Laura López, Diego Castillo, Alfons Torrego

**Affiliations:** ^1^ Pneumology Department Hospital de la Santa Creu i Sant Pau Barcelona Spain; ^2^ Pathological Anatomy Department Hospital de la Santa Creu i Sant Pau Barcelona Spain

**Keywords:** COVID‐19, sarcoidosis, SARS‐CoV‐2

## Abstract

The COVID‐19 pandemic has been a worldwide medical challenge. Despite rapid advancements, many questions regarding SARS‐CoV‐2 interaction with other pathologies and long‐term consequences remained unanswered. Sarcoidosis is a multi‐systemic granulomatous disease that develops in genetically predisposed individuals following their exposure to an environmental trigger. We present the case of a patient who was diagnosed with sarcoidosis following a SARS‐CoV‐2 infection.

## Graphical abstract

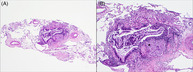

## INTRODUCTION

Despite rapid advancements in the knowledge of COVID‐19, there are still many unanswered questions regarding its possible mid‐to‐long‐term consequences. We present the case of a COVID‐19 patient diagnosed with sarcoidosis after persistent symptoms and pulmonary infiltrates. Some studies have described the increase of infections due to SARS‐CoV‐2 infections in patients with prior diagnoses of sarcoidosis associated, in most cases, with immunosuppressive therapy.^1^, ^2^ However, until the present, no cases of sarcoidosis diagnosis following COVID‐19‐associated disease have been published.

## CASE REPORT

**FIGURE 1:**
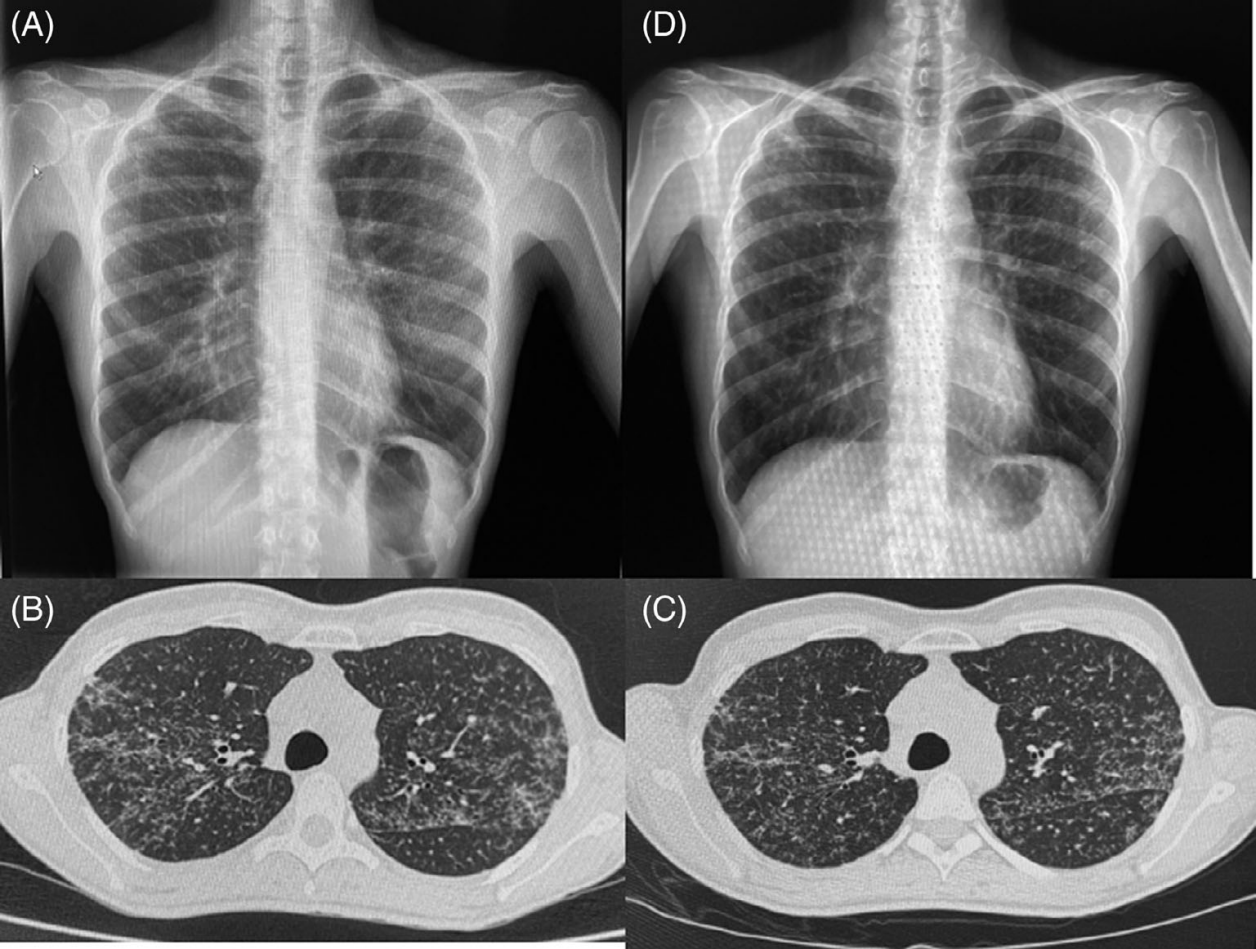
(A) Chest x‐ray performed in March 2020 showing discrete diffusely distributed and bilateral pulmonary infiltrates. (b) Chest computed tomography (CT) performed in May 2020 objectifying diffuse and bilateral micronodular pulmonary infiltrates, predominantly in the upper lobes. (C) Chest CT performed in November 2020 showing no radiological changes. (D) Chest x‐ray performed in June 2021 showing stability of pulmonary infiltrates

We present the case of a 45‐year‐old female patient without any relevant pathological history who came in March 2020 for fever and cough. In the emergency department, SARS‐CoV‐2 test was performed resulting positive. She was hospitalized due to bilateral pulmonary infiltrates detected with a chest x‐ray (Figure 1A). She did not require oxygen therapy and was discharged 24 h later.

Fourteen days later, the patient came again for persistence of cough and the appearance of erythematous cutaneous lesions of 2–3 cm, painful on palpation and localized in the lateral and lower regions of both lower limbs. The persistence of bilateral pulmonary infiltrates was notable in her chest x‐ray. The case was suggestive of post‐infectious cough and cutaneous lesions compatible with recent SARS‐CoV‐2 infection. The patient consulted again 2 weeks later due to persistence of cough, but with complete resolution of cutaneous lesions. A chest computed tomography (CT) was taken, which revealed a parenchymatous bilateral lung infiltrates of diffusely distributed micronodular areas predominantly in the upper lobes and peribronchovascular and interlobar fissures (Figure 1B). Inhaled corticosteroids were initiated for 1 month, and the case was referred to our post‐COVID control unit.

**FIGURE 2:**
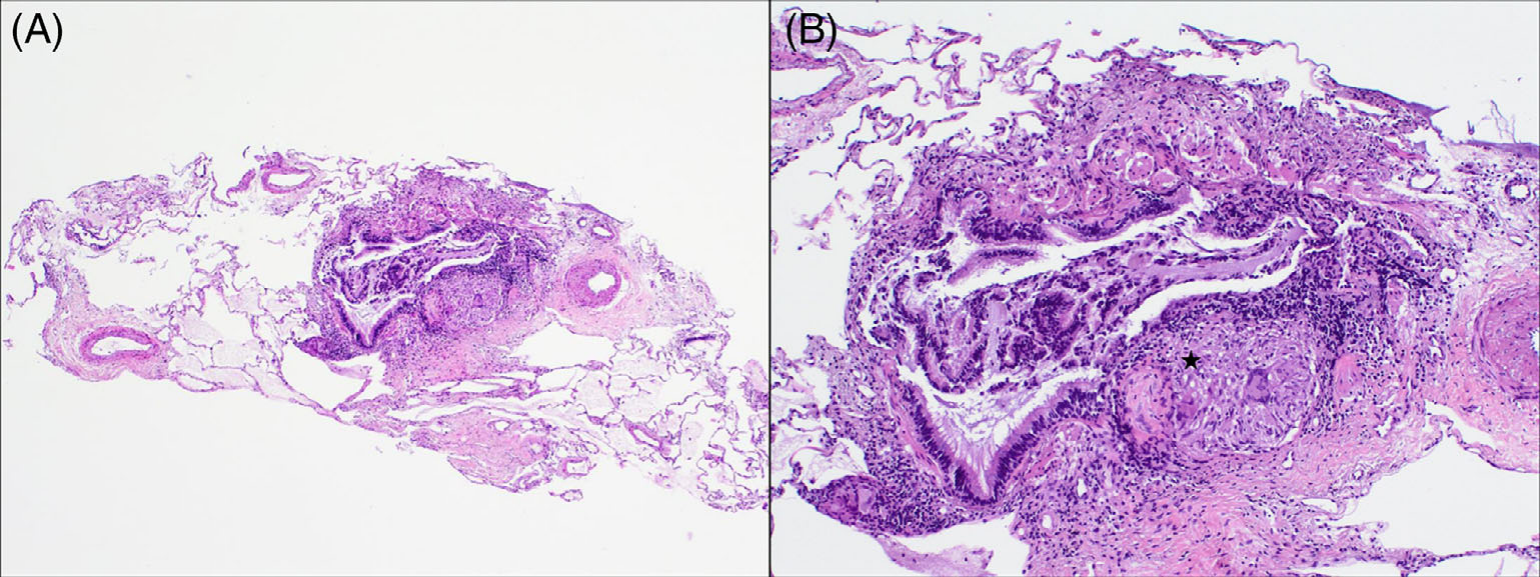
(A) Four fragments of lung tissue were harvested with alveolar parenchyma, without significant microscopic alterations and occasional bronchovascular axes (H&E, 40×). (B) Non‐necrotizing granuloma (star) was observed made up of epithelioid histiocytes, occasional multinucleated giant cells and an associated surrounding lymphocytic component (H&E, 100×)

By August 2021, the patient presented a complete resolution of the cough and normal pulmonary function test. A control chest CT was performed in November 2020, without pulmonary changes (Figure 1C). Considering the radiological findings, further studies were performed. High blood Angiotensin‐converting enzyme (ACE) level (57 nmol/ml/min) as well as CD4/CD8 ratio of 6.4 in bronchoalveolar lavage were observed. A transbronchial cryobiopsy was performed revealing peribronchial non‐necrotizing granuloma (Figure 2). Sarcoidosis diagnosis was established. After diagnosis, a new cutaneous lesion appeared on her upper right limb and the histology also showed granulomatous dermatitis.

At present, the patient is clinically asymptomatic, with radiological (Figure 1D) and functional respiratory stability. Therefore, no oral corticosteroid or immunosuppressive treatment has been initiated.

## DISCUSSION

The authors believe that the case described constitutes a diagnostic challenge with relevant aspects. One important item is the difficulty of diagnosing sarcoidosis following SARS‐CoV‐2 infection. COVID‐19 is a great mimicker and the delayed access to additional examinations is due to the pandemic situation. Persistence of clinical symptoms and the pulmonary infiltrates could have been attributed to post‐COVID sequelae. However, the indication of bronchoscopy and performance of transbronchial cryobiopsy enabled the establishment of a definitive sarcoidosis diagnosis.

On one hand, the similarity between the initial chest x‐ray and the final image (shown in Figure 1), added to the lack of prior radiological studies, could suggest that the sarcoidosis diagnosis was an incidental finding during the SARS‐CoV‐2 infection. Nonetheless, upon evaluation of the case time‐line, a hypothesis could be formed on the role of the SARS‐CoV‐2 infection as a trigger agent for the development of sarcoidosis. As a supporting argument for this hypothesis, several authors have shown the presence of infectious agents that could intervene in its aetiology. Celada et al. performed molecular, genetic and immunological studies on patients with sarcoidosis, proving the roles of a number of infectious agents in the aetiology of sarcoidosis.^3^ Furthermore, two recently published studies have demonstrated, by evaluating the genes involved in the pathogenesis of sarcoidosis and the interactome of the SARS‐CoV‐2 host cell, that the two diseases may involve common cellular pathways in the regulation of autophagy and mitophagy, specifically TBK1 and BRD4.^4^, ^5^

Also noteworthy is the diagnostic interpretation of the cutaneous lesions at the time of SARS‐CoV‐2 infection. In this case, these lesions were initially attributed to the SARS‐CoV‐2 infection. However, in hindsight and having established the final diagnosis, it is possible to consider that these lesions were cutaneous manifestations of sarcoidosis.

The case presented also has certain limitations that hinder confirmation of the role of SARS‐CoV‐2 infection as a trigger factor. The main limitation is the lack of imaging tests prior to COVID‐19. Another is the lack of studies that confirm the definitive participation of COVID‐19 as a trigger factor of other inflammatory or infectious diseases. However, the absence of cases like that described here could be attributed to the difficulty of diagnosis of diffuse pulmonary infiltrates that require invasive techniques to establish a definitive diagnosis. In our case, the COVID‐19 pandemic delayed the performance of additional examinations that, had they been indicated at the appropriate time, would have enabled an earlier diagnosis of the disease, thus avoiding many of the consultations in the emergency department.

## CONFLICT OF INTEREST

None declared.

## AUTHOR CONTRIBUTION

Esther Palones, Alfons Torrego and Virginia Pajares took part in diagnostic techniques and the drafting and supervision of this article. Laura López participated in histological analysis and the authorizations for images related with the case. Diego Castillo contributed to the clinical monitoring of the patient and drafting of this article.

## ETHICS STATEMENT

The authors declare that appropriate written informed consent was obtained for the publication of this manuscript and accompanying images.

## Data Availability

The data that support the findings of this study are available on request from the corresponding author. The data are not publicly available due to privacy or ethical restrictions.
